# Association of Coronavirus Disease 2019 Vaccination with Facial-Related Neurological Disorders: A Nationwide Retrospective Cohort Study

**DOI:** 10.3390/jpm14070671

**Published:** 2024-06-21

**Authors:** Younggoo Kim, Min-Ho Kim, Eunmi Chun, Dosang Cho

**Affiliations:** 1 Department of Neurosurgery, Mokdong Hospital, College of Medicine, Ewha Womans University, Seoul 07985, Republic of Korea; ygkim@ewha.ac.kr; 2 Informatization Department, Seoul Hospital, College of Medicine, Ewha Womans University, Seoul 07804, Republic of Korea; mino-kim@naver.com; 3 Division of Pulmonary and Critical Care Medicine, Department of Internal Medicine, Mokdong Hospital, College of Medicine, Ewha Womans University, Seoul 07985, Republic of Korea; 4 Department of Neurosurgery, Seoul Hospital, College of Medicine, Ewha Womans University, Seoul 07804, Republic of Korea

**Keywords:** facial palsy, trigeminal neuralgia, hemifacial spasm, mRNA, COVID-19, SARS-CoV-2

## Abstract

Neurological complications after the coronavirus disease 2019 (COVID-19) vaccine administration have been reported. However, the incidence rates of these complications have not been compared in vaccinated and unvaccinated individuals. This study used a nationwide cohort from South Korea to investigate the incidence and prognostic factors of facial-related neurological disorders, such as facial palsy, trigeminal neuralgia, and hemifacial spasms, after COVID-19 vaccination. A population-based cohort design was used to examine data from a randomly selected 50% of the adult population in Seoul, South Korea. Information on demographics, vaccination status, vaccination type, and medical history was collected. The incidence rates and adjusted hazard ratios (aHRs) for facial-related neurological disorders were calculated. This study included 2,482,481 adults, 85.94% of whom were vaccinated. Vaccinated individuals showed a higher incidence of facial palsy, hemifacial spasm, and trigeminal neuralgia than unvaccinated individuals, with significant aHRs of 1.821, 3.203, and 6.621, respectively. Dyslipidemia, female sex, and young age were identified as risk factors for hemifacial spasms and trigeminal neuralgia. This study demonstrates an increased incidence of facial-related neurological disorders after COVID-19 vaccination, particularly among individuals with dyslipidemia and younger women. These findings underscore the need for further investigations into the mechanisms and management of vaccine-related neurological issues.

## 1. Introduction

Coronavirus disease 2019 (COVID-19) is a highly infectious disease caused by the novel coronavirus, severe acute respiratory syndrome coronavirus 2 (SARS-CoV-2). The virus emerged in Wuhan, China, in late 2019, and rapidly spread worldwide. On 30 January 2020, the World Health Organization declared the COVID-19 outbreak a global pandemic [1]. COVID-19 has led to unprecedented public health measures, including national lockdowns, social distancing, and rapid development of vaccines. At the time of writing this paper (January 2024), the COVID-19 pandemic had subsided with an increase in the global vaccination rate after the rapid approval and rollout of several vaccines. These vaccines include adenoviral vector vaccines, such as the Oxford-AstraZeneca (AZD1222) and Jassen/Johnson & Johnson (Ad26.COV2-S) vaccines, and messenger ribonucleic acid (mRNA) vaccines, specifically the Pfizer-BioTech (BNT162b2) and Moderna (mRNA-1273) vaccines. Each vaccine employs the viral spike protein (S) as the primary antigen to trigger an immune response. However, the method by which this antigen is introduced into the body differs across vaccine types. mRNA vaccines use mRNA to instruct human cells to produce the spike protein, which prompts an immune response. Adenoviral vector vaccines deliver genetic material from SARS-CoV-2 using a harmless virus as a carrier, causing cells to produce the spike protein [2,3].

The concept of mRNA vaccines was not first used for COVID-19. However, COVID-19 was the first widespread and successful application of this technology in humans on a global scale. The effectiveness of mRNA vaccines against COVID-19 is well documented [4,5]. However, because mRNA vaccines have a different mechanism of action than conventional vaccines and were urgently granted approval based only on the initial phases of clinical trials without completion of all phases, there are many concerns about the related side effects. In most cases, the side effects are mild to moderate, including fever, chills, and headaches. However, some neurological complications manifest as facial symptoms, such as facial palsy or trigeminal neuralgia.

Several studies have reported a temporal relationship between vaccination and the onset of facial palsy [6,7,8], and cases of trigeminal neuralgia [9,10] following COVID-19 vaccination. However, these studies are mainly case reports and series and have limitations. They did not compare the incidence rates with those of unvaccinated individuals in the same COVID-19 era.

Therefore, we designed this study as a nationwide, population-based, longitudinal study. This study aimed to investigate the incidence and prognostic factors of facial-related neurological disorders that are potentially influenced by the vaccine, such as facial palsy, trigeminal neuralgia, and hemifacial spasm, by comparing vaccinated and unvaccinated individuals in a nationwide cohort from South Korea, using national health insurance data.

## 2. Materials and Methods

### 2.1. Study Design and Population

**Figure 1:**
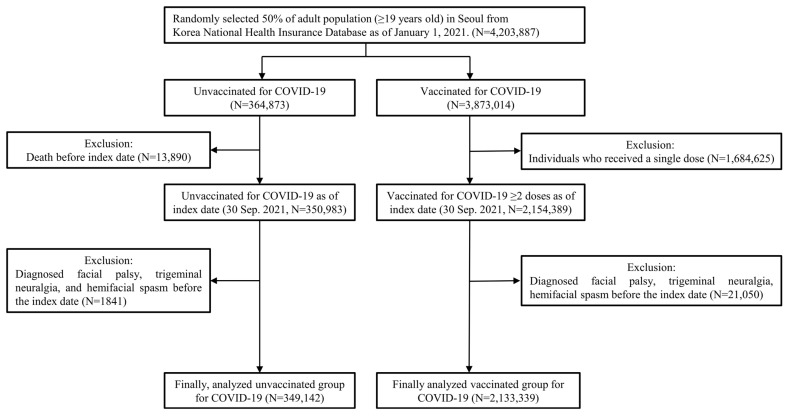
Schematic illustration of participant selection from the Health Insurance Review and Assessment Service database of Korea. COVID-19, coronavirus disease 2019.

This study used a population-based cohort design, examining data from a randomly selected 50% of the adult population (aged ≥19 years) in Seoul (the largest city in South Korea), extracted from the Korea National Health Insurance Database as of 1 January 2021. It used the Korean Classification of Diseases, 7th edition, which is a modified version of the International Classification of Diseases, 10th Edition (ICD-10), for diagnostic coding. The patients were classified into two groups. The first group, regardless of the vaccine type, included individuals who had received two or more doses of the COVID-19 vaccine before 30 September 2021. In this case, the index date was the date of the second vaccination before 30 September 2021. Second, the non-vaccination group was defined as individuals without a single vaccination until the index date (30 September 2021). Participants with primary or secondary diagnoses of vaccination-related diseases, including facial palsy (ICD-10 code G51.0), hemifacial spasm (G51.3), and trigeminal neuralgia (G50.0), i 1 year before the index date were excluded. Vaccination-related diseases were defined as those that occurred when the disease was diagnosed as the primary diagnosis from the day after the index date. Participants with pre-existing facial-related neurological disorders or those who died before the index date were excluded. A schematic illustration of participant selection is shown in Figure 1. This study was approved by the Institutional Review Board (IRB) of Ewha Womans University Mokdong Hospital (IRB No. 2023-12-029). It was conducted according to the ethical standards of the Declaration of Helsinki. The requirement for written informed consent was waived because of the retrospective nature of this study.

### 2.2. Data Collection

From the data of randomly selected patients, information was collected on demographics, such as age and sex, vaccination status and type, primary and secondary diagnoses from 2020 to 2021, dates of hospital visits, underlying diseases, and history of COVID-19. The covariates used were age, sex, insurance premium grade, Charlson Comorbidity Index (CCI), presence of diabetes (E10–E14), hypertension (I10–I13, I15), dyslipidemia (E78), chronic pulmonary disease (J40–J47, J60–J67), and history of COVID-19. The insurance premium grade was categorized into three levels based on health insurance premiums: low, middle, and high. The selection of diagnostic codes for each disease under the CCI was based on the findings of a previous study by Sundararajan et al. [11]. The presence of diseases included in the CCI was defined as registration as a primary or secondary diagnosis more than twice a year before the index date. The status of COVID-19 was determined by the presence of the ICD-10 code U071 in either primary or secondary diagnoses before the index date.

### 2.3. Statistical Analysis

The results are expressed as mean ± standard deviation or the actual number of individuals and their proportions. Student’s *t*-test was used to compare continuous variables. Chi-squared test or Fisher’s exact test was used to compare categorical variables. The incidence rate per 10,000 individuals was then calculated. A multiple logistic regression model was used to estimate the odds ratio (OR) and the corresponding 95% confidence interval (95% CI) and *p*-value. The Cox regression model was used to calculate the hazard ratio (HR) and the corresponding 95% CI and *p*-value. A *p*-value of <0.05 denoted statistical significance. The SAS Enterprise Guide (SAS Institute, Cary, NC, USA) was used for all statistical analyses and data curation.

## 3. Results

### 3.1. Baseline Characteristics

The cohort consisted of 2,482,481 adults, of whom 349,142 (14.06%) were unvaccinated, and 2,133,339 (85.94%) received the COVID-19 vaccination. Men represented 45.20% of the total population (1,122,023), with a slightly higher percentage in the unvaccinated group (45.94%, 160,380) than in the vaccinated group (45.08%, 961,643). The mean age of the entire cohort was 54.85 ± 17.04 years. A distinction between the groups revealed that the unvaccinated individuals were younger, with a mean age of 45.55 ± 17.31 years, whereas the vaccinated individuals were older, averaging 56.38 ± 16.50 years. As shown in Table 1, the vaccinated group had higher insurance levels and more comorbidities than the unvaccinated group. The higher the level of insurance and comorbidities, the more people are interested in their health; thus, more people were likely to have been vaccinated. Within the vaccinated cohort, individuals were classified according to the type of COVID-19 vaccine they received. Most of the 1,227,282 individuals (57.53%) received only mRNA vaccines, 767,247 (35.96%) received only viral vector vaccines, and a smaller subset of 138,810 (6.51%) received cross-vaccination protocols that involved receiving different types of vaccines (Table 1). The vaccination status for different types of vaccines is shown in Supplementary Table S1.

### 3.2. Association between Facial-Related Neurological Disorders and COVID-19 Vaccination

**Figure 2:**
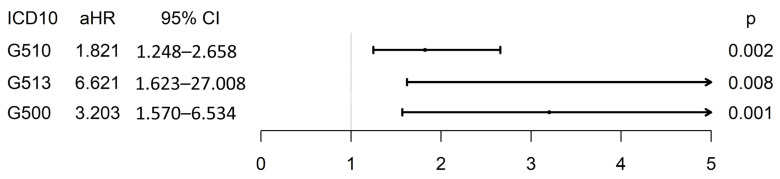
Adjusted hazard ratios (aHRs) for facial palsy, hemifacial spasm, and trigeminal neuralgia. CI, confidence interval; ICD-10, International Classification of Diseases, 10th Edition.

The incidence of facial-related neurological disorders was compared between the vaccinated and unvaccinated groups. The incidence rates did not show statistically significant differences between the two groups 1 month after the index date. However, both groups showed statistically significant differences in the incidence rates at 3 months (Table 2). Adjusted hazard ratios (aHRs) for facial palsy, hemifacial spasms, and trigeminal neuralgia were calculated. The vaccinated group demonstrated a higher incidence of facial palsy (aHR 1.821, 95% CI 1.248–2.658, *p* = 0.002), hemifacial spasms (aHR 3.203, 95% CI 1.570–6.534, *p* < 0.001), and trigeminal neuralgia (aHR 6.621, 95% CI 1.623–27.008, *p* = 0.008) than the unvaccinated group (Figure 2).

### 3.3. Risk Factors for the Development of Facial-Related Neurological Disorders after COVID-19 Vaccination

This study also analyzed the prognostic factors for facial-related neurological diseases observed in the vaccinated group. In the vaccinated group, the risks of facial palsy, hemifacial spasms, and trigeminal neuralgia were increased regardless of the type of vaccine. However, the increased occurrence of facial palsy due to cross-vaccination in patients who developed facial palsy was not statistically significant. In addition, the independent poor prognostic factors related to the occurrence of facial-related neurological disorders in the vaccinated group were female sex and young age for all diseases except facial palsy. Regarding facial palsy, the risk of developing facial-related neurological disorders was lower in women and was not related to age. Dyslipidemia appeared to be a poor prognostic factor for all three diseases. Unlike other diseases, hypertension was also associated with the occurrence of hemifacial spasms (Table 3).

## 4. Discussion

This nationwide, population-based, longitudinal study provides critical insights into the incidence and prognostic factors of facial-related neurological disorders following COVID-19 vaccination. Our finding that the incidence rates of facial palsy, trigeminal neuralgia, and hemifacial spasm were significantly higher in vaccinated individuals is significant because we verified the correlation between these conditions and vaccination using large-scale data beyond the level of case reports. This study also found that dyslipidemia was associated with the development of facial-related neurological disorders after vaccination.

In a retrospective study of 13 patients who developed facial palsy within 42 days after vaccination, Kim et al. reported that COVID-19 vaccines, especially mRNA vaccines, may be correlated with facial palsy with distinct clinical characteristics, which occurs more frequently in young adults and is often accompanied by changes in taste. It showed faster and better recovery compared with vaccine-unrelated facial palsy [12]. In contrast, Shemer et al. reported no increase in the risk of facial paralysis after vaccination in a case–control study involving 37 patients with acute-onset facial paralysis and matched controls [13]. Although conflicting results have been reported, it is widely accepted that facial palsy may occur as an adverse event following vaccination with COVID-19 vaccines, especially mRNA vaccines, based on several recently reported research results [14,15,16]. Unlike facial palsy, trigeminal neuralgia has also been reported as a potential complication of COVID-19 vaccination in case reports. Multiple cases have been documented, including a 36-year-old woman who developed trigeminal neuralgia after receiving the Pfizer BioNTech vaccine [9]. Another case involved a teenage boy who experienced facial nerve paralysis and sensory loss in the trigeminal nerve distribution following the Pfizer-BioNTech vaccination [17]. A case study also described trigeminal neuropathy in a patient who had previously undergone microvascular decompression for trigeminal neuralgia [18]. No studies or case reports have been published regarding the association between hemifacial spasms and COVID-19 vaccination.

As mentioned above, the association between facial-related neurological disorders and COVID-19 vaccination remains unclear. However, our results suggest several possible links among individuals who develop facial-related neurological symptoms after vaccination. This phenomenon may be related to the immune response to the vaccine, potentially triggering an inflammatory process affecting the facial and trigeminal nerves, leading to facial disorders. First, vaccinations elicit an immune response to prepare the body to fight future infections. In rare instances, this response can inadvertently affect the cranial nerves, potentially leading to facial-related neurological disorders. Molecular mimicry is one mechanism by which this may occur. The immune system may confuse the proteins in nerve cells with those from the vaccine antigen, leading to an attack on the nerves. The molecular mimicry between SARS-CoV-2 and human proteins, including those in the nervous system, is a concern regarding COVID-19 vaccine use. The spike protein of SARS-CoV-2, which is used in vaccine development, may share sequences with proteins involved in myelin and axon homeostasis, potentially leading to autoimmune reactions against the nervous system [19]. Second, immune complexes, which are formed when antibodies bind to multiple antigens [20], could be deposited in the nerve sheath, leading to inflammation and subsequent nerve damage. These complexes, composed of autoantibodies and nucleic acids, induce complement activation and tissue injury in various organs [21]. Studies have shown that immune complexes can form lattice structures deposited in different tissues, causing cellular damage and clinical manifestations of immune complex disease [22]. The constituents of immune complexes, including the antibody isotype and antigen, play crucial roles in the pathology associated with immune complex deposition in tissues. Overall, the immune-complex-mediated inflammation of nerve sheaths can result in significant nerve damage, highlighting the importance of understanding and potentially targeting these processes in therapeutic interventions.

Interestingly, there was no statistically significant difference in facial-related neurological symptoms between the two groups at 1 month after vaccination. However, there was a statistically significant difference between the two groups at 3 months. This is likely due to the adaptive immune system rather than the innate immune system, such as anaphylaxis, where symptoms occur immediately after vaccination.

As can be seen from the results of this study, dyslipidemia was associated with the development of facial-related neurological diseases after vaccination. Dyslipidemia is associated with various immune responses. Studies have shown a positive correlation between the systemic-immune-inflammation index and dyslipidemia, indicating a potential link between inflammation and lipid levels [23]. Dyslipidemia can enhance inflammatory responses in diseases such as COVID-19, and dysregulated lipid metabolism in a dyslipidemic environment can affect autoimmune T-cell responses, particularly T helper 17 cells and T follicular helper cells, which play a role in dyslipidemia-associated autoimmune diseases [24].

Hemifacial spasm and trigeminal neuralgia were more prevalent in women and younger age groups, whereas facial palsy appeared to be less prevalent in women and was not associated with age. Unlike other conditions, hypertension appeared to be a risk factor for hemifacial spasms. Further research is needed to understand how facial palsy differs between patients with hemifacial spasms and those with trigeminal neuralgia. In contrast to several case reports highlighting symptoms such as facial palsy after mRNA vaccination, the present study showed that the risk of facial-related neurological disorders was higher with conventional vaccines or a combination of both vaccines than with mRNA vaccines alone. Therefore, more extensive and well-designed studies on the safety and adverse effects of mRNA vaccines are needed instead of vague concerns about them.

The most significant limitation of this study is that it cannot guarantee the validity of the diagnosis and does not reflect disease severity. We defined facial-related neurological disorders based on ICD codes from insurance claims data. However, numerous studies using claims-based definitions have been reported, and these administrative data have high specificity with variable sensitivity to diagnosis and medical conditions [25]. These disorders may have been underestimated because we used insurance data based on hospital visits. In addition, Seoul is the largest city in Korea and is home to 20% of the Korean population. However, the study’s results do not represent Korea as a whole. Moreover, these results are difficult to generalize because the medical environments in Korea and overseas differ. Despite these limitations, this study is significant because it is the first to examine the relationship between facial-related neurological disorders and COVID-19 using national claims-based data.

## 5. Conclusions

This nationwide study indicated an increased incidence of facial palsy, trigeminal neuralgia, and hemifacial spasms following COVID-19 vaccination, particularly among individuals with dyslipidemia and younger women. These findings suggest the need for further research to clarify these associations and understand the underlying mechanisms, particularly the role of immune responses and vaccine types in the development of these neurological disorders. Additional studies are needed to improve vaccine safety and management strategies for affected individuals.

## Figures and Tables

**Table 1:** Demographic characteristics of the study population according to vaccination status.

Variables	Total	Unvaccinated Group	Vaccinated Group	*p*-Value
Total	2,482,481	349,142 (14.06)	2,133,339 (85.94)	
Sex				
Male	1,122,023 (45.20)	160,380 (45.94)	961,643 (45.08)	<0.001
Female	1,360,458 (54.80)	188,762 (54.06)	1,171,696 (54.92)	
Age (years)	54.85 ± 17.04	45.55 ± 17.31	56.38 ± 16.50	<0.001
20–29	273,137 (11.00)	64,066 (18.35)	209,071 (9.80)	<0.001
30–39	252,600 (10.18)	89,142 (25.53)	163,458 (7.66)	
40–49	303,535 (12.23)	72,953 (20.89)	230,582 (10.81)	
50–59	578,648 (23.31)	49,405 (14.15)	529,243 (24.81)	
60–69	587,067 (23.65)	36,284 (10.39)	550,783 (25.82)	
70–79	326,823 (13.17)	18,305 (5.24)	308,518 (14.46)	
80–	160,671 (6.47)	18,987 (5.44)	141,684 (6.64)	
Insurance level				
Low	643,906 (25.94)	102,271 (29.29)	541,635 (25.39)	<0.001
Middle	678,694 (27.34)	108,818 (31.17)	569,876 (26.71)	
High	1,159,881 (46.72)	138,053 (39.54)	1,021,828 (47.90)	
CCI				
0	1,596,505 (64.31)	286,873 (82.17)	1,309,632 (61.39)	<0.001
1	437,370 (17.62)	28,166 (8.07)	409,204 (19.18)	
2≤	448,606 (18.07)	34,103 (9.77)	414,503 (19.43)	
Comorbidity				
Diabetes	420,238 (16.93)	24,987 (7.16)	395,251 (18.53)	<0.001
Dyslipidemia	845,395 (34.05)	46,557 (13.33)	798,838 (37.45)	<0.001
Hypertension	736,141 (29.65)	41,047 (11.76)	695,094 (32.58)	<0.001
Chronic pulmonary disease	114,560 (4.61)	9083 (2.60)	105,477 (4.94)	<0.001
COVID-19 history	20,847 (0.84)	4176 (1.20)	16,671 (0.78)	<0.001
Type of vaccine				
No vaccination	349,142 (14.06)	349,142 (100)		
Only mRNA vaccine	1,227,282 (49.44)		1,227,282 (57.53)	
Only viral vector vaccine	767,247 (30.91)		767,247 (35.96)	
Cross-vaccination	138,810 (5.59)		138,810 (6.51)	

CCI, Charlson Comorbidity Index; COVID-19, coronavirus disease 2019; mRNA, messenger ribonucleic acid.

**Table 2:** Incidence of newly diagnosed facial-related neurological disorders following COVID-19 vaccination.

Disease	Vaccination	Total	1 Month				3 Months			
			Events	IR	95% CI	*p*-Value	Events	IR	95% CI	*p*-Value
Facial palsy (G51.0)	No	349,142	13	0.37	0.17–0.57	0.115	30	0.86	0.55–1.17	<0.001
	Yes	2,133,339	129	0.60	0.50–0.71		417	1.95	1.77–2.14	
Hemifacial spasm (G51.3)	No	349,142	0	0.00	0.00–0.00	0.065	2	0.06	0.00–0.14	<0.001
	Yes	2,133,339	23	0.11	0.06–0.15		98	0.46	0.37–0.55	
Trigeminal neuralgia (G50.0)	No	349,142	6	0.17	0.03–0.31	0.229	8	0.23	0.07–0.39	<0.001
	Yes	2,133,339	63	0.30	0.22–0.37		206	0.97	0.83–1.10	

IR, incidence rate; CI, confidence interval; COVID-19, coronavirus disease 2019.

**Table 3:** Prognostic factors for the development of facial-related neurological disorders 3 months after vaccination.

Variables	Facial Palsy (G51.0)			Hemifacial Spasm (G51.3)			Trigeminal Neuralgia (G50.0)		
	HR	95% CI	*p*-Value	HR	95% CI	*p*-Value	HR	95% CI	*p*-Value
Vaccine									
No vaccination	1.000	Reference		1.000	Reference		1.000	Reference	
Only mRNA vaccine	1.814	1.233–2.668	0.003	4.913	1.181–20.437	0.029	2.842	1.375–5.874	0.005
Only viral vector vaccine	1.934	1.293–2.892	0.001	9.156	2.193–38.235	0.002	3.797	1.821–7.918	<0.001
Cross-vaccination	1.594	0.904–2.809	0.107	9.970	2.113–47.049	0.004	3.708	1.513–9.086	0.004
Sex, female	0.804	0.667–0.970	0.022	2.522	1.590–4.001	<.001	1.367	1.034–1.807	0.028
Age	1.005	0.998–1.012	0.173	1.022	1.005–1.038	0.010	1.013	1.002–1.024	0.017
Insurance level									
Low	1.000	Reference		1.000	Reference		1.000	Reference	
Middle	1.006	0.778–1.300	0.966	1.609	0.887–2.917	0.117	1.203	0.830–1.744	0.329
High	0.988	0.788–1.239	0.919	1.712	0.903–1.922	0.078	1.080	0.773–1.507	0.652
CCI									
0	1.000	Reference		1.000	Reference		1.000	Reference	
1	1.197	0.901–1.590	0.215	1.124	0.645–1.959	0.680	1.180	0.789–1.763	0.420
2≤	1.215	0.880–1.679	0.237	0.804	0.397–1.628	0.545	1.640	1.071–2.512	0.023
Diabetes	1.211	0.915–1.603	0.181	0.638	0.315–1.291	0.212	0.827	0.559–1.225	0.344
Hypertension	0.898	0.709–1.136	0.368	0.525	0.310–0.887	0.016	0.819	0.587–1.142	0.239
Dyslipidemia	1.458	1.137–1.868	0.003	1.699	1.271–2.116	0.037	1.488	1.055–2.101	0.024
Chronic pulmonary disease	1.223	0.839–1.784	0.295	1.509	0.656–3.468	0.333	1.291	0.782–2.132	0.318
COVID-19 history	0.803	0.112–5.745	0.827	Not available			0.513	0.128–2.058	0.346

HR, hazard ratio; CI: confidence interval; mRNA, messenger ribonucleic acid; CCI: Charlson Comorbidity Index; COVID-19, coronavirus disease 2019.

## Data Availability

The data presented in this study are available on request from the corresponding authors.

## Supplementary Materials

The following supporting information can be downloaded at: https://www.mdpi.com/article/10.3390/jpm14070671/s1, Table S1: Number and ratio of vaccinated people by vaccine type.
