# Supplementary material for: Association of Coronavirus Disease 2019 Vaccination with Facial-Related Neurological Disorders: A Nationwide Retrospective Cohort Study
Source: J Pers Med. 2024 Jun 21;14(7):671. doi: 10.3390/jpm14070671 (PMC11277844; doi:10.3390/jpm14070671)
Supplement: Supplementary file 1 [file jpm-14-00671-s001.zip › jpm-3053525-supplementary.pdf]

Supplementary Table S1. Number and ratio of vaccinated people by vaccine type

Variables	Total<br>2,133,339 (100%)
Type of vaccine	
Only mRNA vaccine	1,227,282 (57.52%)
1st Pfizer – 2nd Pfizer	1,196,362 (56.07%)
1st Pfizer – 2nd Moderna	35 (0.00%)
1st Moderna – 2nd Pfizer	9 (0.00%)
1st Moderna – 2nd Moderna	30,876 (1.48%)
Only viral vector vaccine	767,247 (35.96%)
1st AstraZeneca – 2nd Jassen	7 (0.00%)
1st AstraZeneca – 2nd AstraZeneca	767235 (35.96%)
1st Jassen– 2nd AstraZeneca	5 (0,00%)
1st Jassen – 2nd Jassen	0 (0.00%)
Cross	138,810 (6.51%)
1st Pfizer – 2nd AstraZeneca	6 (0.00%)
1st Pfizer – 2nd Jassen	3 (0.00%)
1st AstraZeneca – 2nd Pfizer	138791 (6.51%)
1st AstraZeneca – 2nd Moderna	3 (0.00%)
1st Jassen – 2nd Pfizer	7 (0.00%)
